# Individualized Total Knee Arthroplasty Using the Origin^®^: A Step-by-Step Surgical Technique and Clinical Application

**DOI:** 10.3390/jcm15093356

**Published:** 2026-04-28

**Authors:** Mohammad Alajji, Marc Barrera Uso, Axel Schmidt, Thais Dutra Vieira, Tarik Ait Si Selmi, Michel Bonnin, Elliot Sappey-Marinier

**Affiliations:** Centre Orthopedique Santy, Ramsay Sante, Hôpital Prive Jean Mermoz, 69008 Lyon, France

**Keywords:** total knee replacement, individualized total knee arthroplasty, knee, Origin^®^ individualized implants

## Abstract

**Background:** Total knee arthroplasty (TKA) is a common procedure aimed at alleviating knee pain and restoring function in patients with degenerative joint diseases. Traditional implants are typically designed to restore mechanical knee alignment, but personalized implants have shown promise in improving clinical outcomes. The Origin^®^ individualized TKA system provides a tailored approach to knee reconstruction by utilizing preoperative 3D planning to create individualized implants and cutting guides based on each patient’s unique anatomy. **Surgical Technique:** The Origin^®^ system employs a preoperative computed tomography (CT) scan and Knee-Plan^®^ software to design individualized implants that optimize alignment and joint anatomy. The surgical technique involves the use of patient-specific cutting guides for precise bone resections and the insertion of either cruciate-retaining (CR) or posterior-stabilized (PS) implants, depending on individual patient needs. This process aims to replicate the pre-arthritic alignment and kinematics of the pre-arthritic knee. **Postoperative Protocol:** The postoperative protocol allows for immediate weight-bearing, and patients are guided through a structured rehabilitation program to ensure optimal recovery. Full range-of-motion exercises begin early to promote knee mobility and strength. **Discussion:** The individualized TKA system offers several advantages, including precise restoration of pre-arthritic anatomy, reduced bone resection, and improved implant fit. These benefits are particularly valuable in patients with unique anatomical challenges, such as deformities or previous surgeries. Despite the potential advantages, challenges remain, including the costs and time associated with individualized manufacturing, as well as increased radiation exposure from the required CT scans. **Conclusions:** The Origin^®^ individualized TKA system represents a significant advancement in knee arthroplasty by providing a tailored approach to patient care. Future studies are needed to further evaluate the long-term outcomes and cost-effectiveness of this personalized system compared to conventional TKA approaches.

## 1. Introduction

Total knee arthroplasty (TKA) is one of the most commonly performed orthopedic procedures worldwide and represents a highly effective treatment for end-stage knee osteoarthritis [1]. Historically, TKA has been based on the principle of restoring a neutral mechanical alignment, aiming for a hip–knee–ankle axis of approximately 180°, with the goal of optimizing implant longevity and load distribution. However, despite excellent survivorship, a substantial proportion of patients (up to 20% in some series) remain dissatisfied after surgery, prompting a paradigm shift toward more individualized approaches to knee reconstruction [2,3,4,5].

In recent years, increasing evidence has challenged the concept of a “one-size-fits-all” alignment strategy, highlighting the wide variability in native knee anatomy and alignment among individuals. This has led to the development of several alternative alignment philosophies aimed at restoring a more physiological joint configuration. Among these, kinematic alignment (KA) seeks to reproduce the patient’s pre-arthritic joint lines and ligament laxities, thereby restoring native knee kinematics. Variations in this concept include restricted kinematic alignment (rKA), which applies boundaries to avoid excessive deviations from neutral alignment in extreme anatomies, and inverse kinematic alignment (iKA), which prioritizes tibial resurfacing before femoral adjustments. Other alignment strategies have also been proposed, including anatomical alignment (AA), which aims to recreate the native joint line obliquity while maintaining overall limb alignment within safe limits, and functional alignment (FA), a more recent, technology-driven approach that combines patient-specific anatomy with intraoperative soft-tissue balancing, often facilitated by computer navigation or robotic assistance, to achieve an individualized yet controlled alignment [2]. Collectively, these approaches reflect a broader transition toward personalized TKA, in which implant positioning, alignment, and soft-tissue management are tailored to the individual patient rather than standardized targets.

Parallel to the evolution of alignment philosophies, advances in imaging, digital planning, and manufacturing technologies have enabled the development of patient-specific solutions. These include patient-specific instrumentation (PSI), which uses preoperative imaging to guide bone cuts, and fully customized implants, designed to match each patient’s unique anatomy. While PSI improves surgical accuracy, it does not modify implant geometry. In contrast, individualized implants aim to reproduce the native morphology of the femur and tibia, potentially improving implant fit, restoring joint line orientation, and enhancing kinematic behavior. In this context, individualized implants, which are specifically designed based on each patient’s anatomy and manufactured on demand, aim to maximize clinical outcomes by closely replicating pre-arthritic morphology and alignment [6].

The Origin^®^ individualized TKA (Symbios, Yverdon-les-Bains, Switzerland) has been specifically conceived to optimize knee kinematics and joint function through a fully personalized approach. Using preoperative three-dimensional planning, the system enables precise alignment, minimal bone resection, and high reproducibility. Both cruciate-retaining (CR) and posterior-stabilized (PS) configurations are available, allowing adaptation to patient-specific stability requirements. This paper outlines the surgical technique for both systems and compares their indications and clinical applications within the evolving landscape of personalized knee arthroplasty.

## 2. Surgical Technique

### 2.1. Preoperative Planning

The Origin^®^ system features a preoperative planning interface built on the patient’s computed tomography (CT) scan, using a dedicated protocol. Based on these scans, individualized implants and cutting guides are custom-manufactured over approximately six weeks and delivered in a single package to the hospital. Preoperative planning is carried out on a three-dimensional CT-based reconstruction using the Knee-Plan^®^ software (Version 2.0.FR), which automatically calculates overall limb alignment (hip-knee-ankle axis), joint line obliquity, posterior tibial slope, and the main rotational axes, while also quantifying the location and depth of bone wear in each compartment. Based on these measurements, the software proposes individualized femoral and tibial components and patient-specific cutting guides that reproduce the native bone contours and joint line orientation; the surgeon then reviews and, when necessary, fine-tunes the realignment strategy before final validation, following an rKA philosophy. In this context, “pre-arthritic anatomy” is defined as the hypothetical native morphology and alignment of the knee before cartilage loss, osteophyte formation, and secondary bone remodeling. Practically, this is estimated by virtually removing osteophytes and deforming bone overgrowth from the 3D model and by accounting for the quantified bone wear to restore the presumed original joint line level and condylar radii, while keeping the patient’s constitutional coronal, sagittal, and axial alignment within the safety limits of rKA.

Once the planning is confirmed, definitive implants, including a chromium-cobalt femoral component and a titanium tibial baseplate, are manufactured to mirror the native pre-arthritic bone shape, along with the production of custom polyamide cutting guides. It is possible to add a titanium tibial stem ranging from 20 mm to 70 mm, which needs to be decided at the preoperative phase. It is recommended to add a 20 to 30 mm tibial stem for PS implants. In patients with severe deformities or advanced osteoarthritis, the preoperative plan is used to simulate different correction strategies and implant positions so that the final reconstruction represents a compromise between restoring the pre-arthritic joint line and condylar morphology and maintaining mechanical safety in terms of bone stock preservation, implant fixation, and soft-tissue balance.

### 2.2. Patient Setup

The patient is positioned supine on the operating table, with a foot roll to allow flexion and extension of the knee along its range of motion (ROM) during the whole procedure and a lateral support to avoid external rotation of the hip.

### 2.3. Joint Exposure

The skin incision is made with the knee flexed or extended based on the surgeon’s preference. Then, a medial parapatellar approach is performed, and the knee joint is exposed, with careful excision of the Hoffa’s infrapatellar pad and anterior cruciate ligament (ACL). Soft-tissue releases are kept to a minimum. Beyond the standard medial parapatellar approach, only a limited deep release of the medial collateral ligament is performed, just enough to expose the medial tibial plateau and allow correct positioning of the cutting guides and implants, as in a conventional TKA. No additional posteromedial, lateral, extensive capsuloligamentous releases or medial collateral ligament pie crust are performed routinely. If a CR implant has been planned, a complete preservation of the posterior cruciate ligament (PCL) is mandatory. If a PS system is to be implanted, the PCL should be completely resected. Joint exposure should be wide enough to allow for stable positioning of both the femoral and tibial cut guides.

### 2.4. Femur Preparation

**Figure 1:**
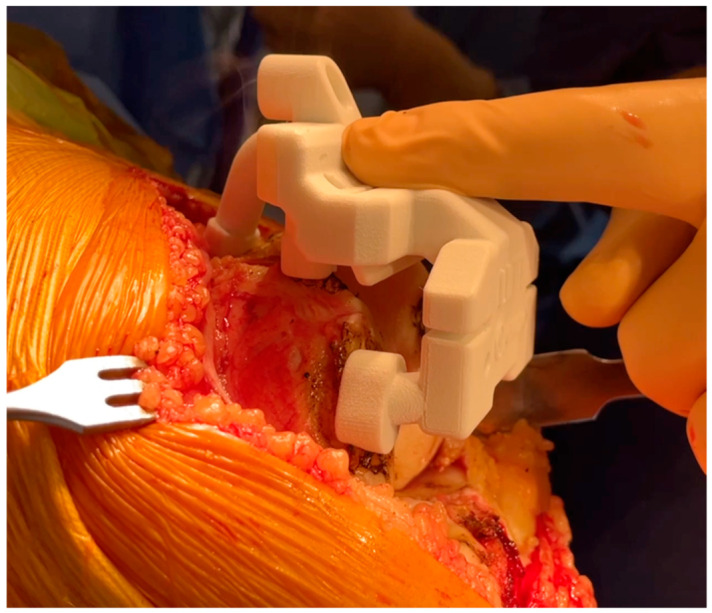
Custom cutting guide placement on the femur for precise bone resection.

**Figure 2:**
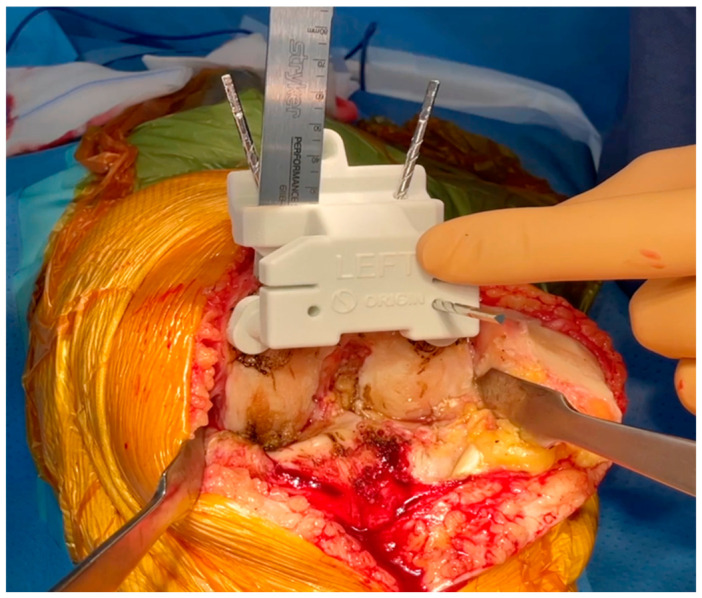
Pin fixation and positioning of the custom femoral cutting guide.

**Figure 3:**
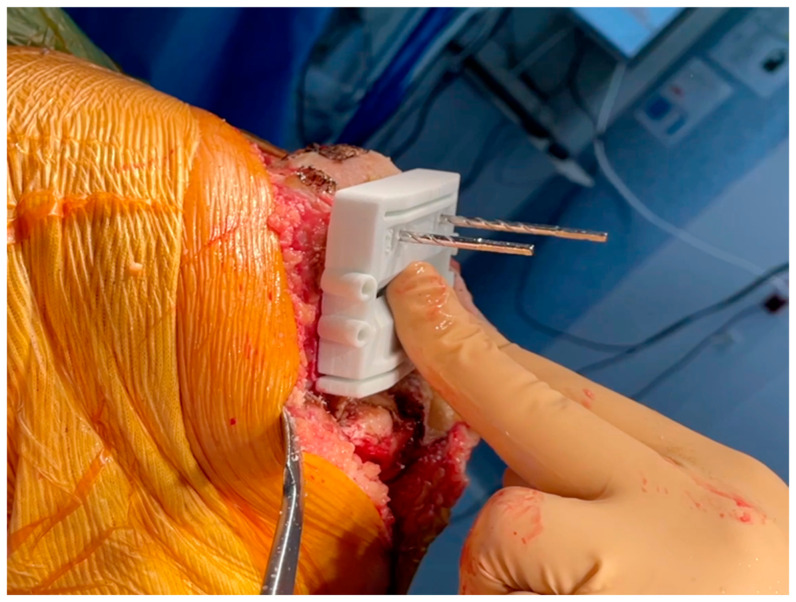
Verification positioning of the femoral guide (4in1) to ensure proper alignment.

Once the femur is exposed, the femoral preparation is done first, because the femur is the driver of the knee kinematics. The first surgical step is to remove the remnants of cartilage, using electrocautery, a curette, or a scalpel blade, in the areas of contact of the cutting block. The first femoral guide is secured to the bone with pins as soon as a unique and stable position has been found for it, and cuts are performed with an oscillating saw (Figure 1 and Figure 2). No femoral recuts are needed, as the aim of the procedure is to reproduce precisely the shape of the distal condyles. The ‘four in one’ femoral cutting guide (second femoral guide) is positioned on the distal resected femur without any adjustment in size or rotation (Figure 3). The concept of femoral rotation has here no meaning because the femoral implant reproduces the shape of the distal femur, and the thickness of the polyethylene reproduces the native joint line obliquity. For the PS system, resection of the intercondylar femoral notch is guided by the third femoral guide. The medio-lateral contour of the bloc—specific to the patient—matches the bony contours of the femur. The trial femoral component is positioned on the distal femur, and flexion/extension motion and valgus/varus stressing are done to assess the amount of bone wear and the level of the tibia cut.

### 2.5. Tibial Preparation

**Figure 4:**
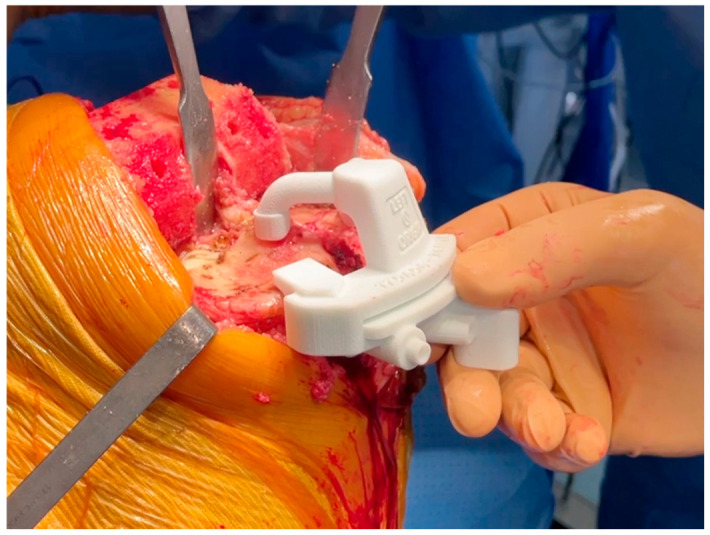
Tibial cutting guide placement for accurate tibial resection.

After removal of the cartilage remnants, the tibial guide is positioned on the plateaus and secured with pins once the position is stable (Figure 4). To meet the planned cut’s orientation, the extramedullary alignment control rod must be centered medio-laterally to the center of the ankle joint. The resection provided by the tibial guide is a minimal cut with respect to the planning. In some cases, a recut must be done as a second step (corresponding to the planned resection).

**Figure 5:**
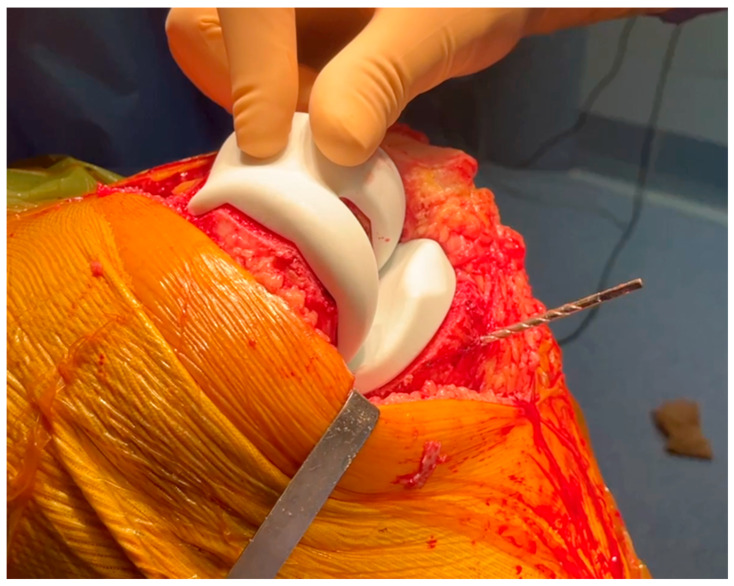
Trial implant placement to assess fit and stability before final implantation.

The trial femoral component was then positioned (Figure 5), and ligament balancing was tested with an 8 mm trial insert (labeled ‘+2 mm’), which simulates the thickness of a standard 6 mm polyethylene insert (labeled ‘0 mm’) mounted on the tibial baseplate. The mediolateral stability at full extension and mid-flexion and the full range of motion were evaluated. After testing, three situations could be observed:

Well balanced: When a full range of motion is achieved, the final implants should be cemented with the standard 6 mm polyethylene insert.

Stiff: Ligament balance should be reassessed using a 6 mm trial insert (labeled “0”) to simulate a 2 mm tibial recut. When balance is optimal with the 6 mm insert, a 1 mm or 2 mm tibial recut should be performed using the millimetric re-cutting guide. The final implants should be cemented with the standard 6 mm polyethylene insert.

Lax: Ligament balance should be reassessed using the trial tibial baseplate and an 8 mm trial insert (labeled “+2 mm”). When balance is satisfactory, the final implants should be cemented with an 8 mm polyethylene insert (labeled “+2 mm”).

After obtaining a good range of motion, with a balanced knee, the definitive tibial preparation is performed. The custom tibial baseplate (keel position and contouring are patient-specific) is then fixed on the resected tibia surface, and the central peg and fins are prepared.

### 2.6. Patellar Preparation (Optional)

Following patellar exposure and resection of marginal osteophytes, the patella is secured into the cutting clamp until it is firmly stable, and the resection height is adjusted, leaving at least 13 mm of bone thickness. The patella is then cut with an oscillating saw parallel to the anterior cortical bone, and the component size is determined based on the preoperative planning or using available trial components. Once the appropriate size is selected, a compression clamp is positioned, stabilized with a drill pin, and anchoring pin holes are drilled.

### 2.7. Final Implantation

**Figure 6:**
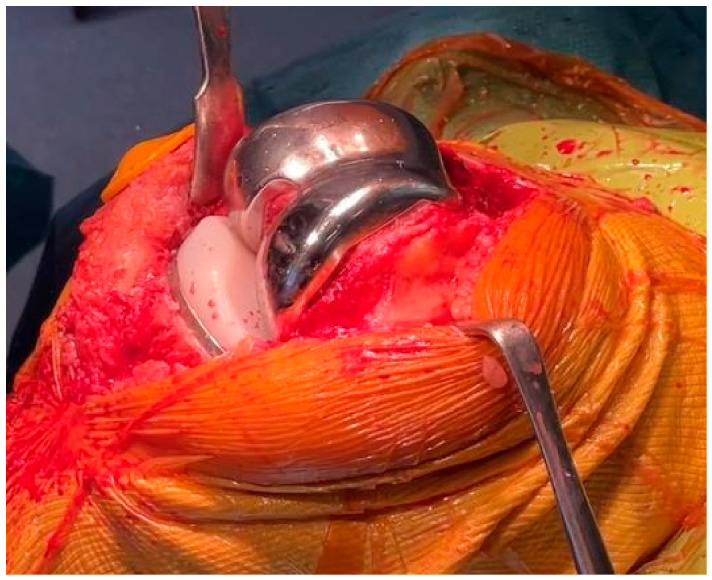
Final implantation of the custom total knee arthroplasty components.

**Figure 7:**
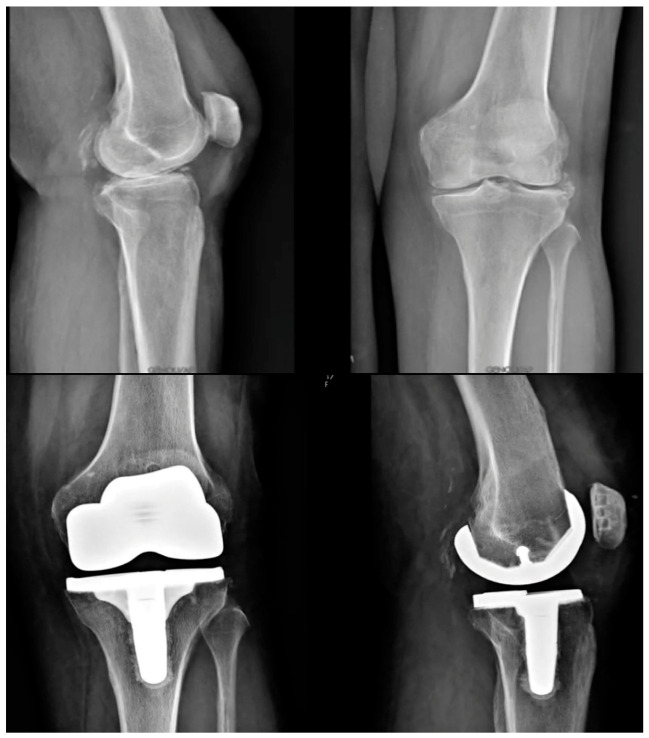
Preoperative (**top**) and postoperative (**bottom**) X-ray images of a patient undergoing individualized TKA with the Origin^®^ system.

The trochlea of the Origin^®^ prosthesis is designed to match the shape of the native patella (anatomic trochlea), so patellar resurfacing is not required but is recommended in cases of severe patellar osteoarthritis (i.e., Iwano stages 3–4 [7]). In case of trochlea dysplasia, the abnormal trochlea is not recreated, and a prosthetic trochlea is created matching the shape of the patellar button. Therefore, in case of preoperative trochlea dysplasia, it is recommended to resurface the patella. Once all bone surfaces have been prepared, the implants are cemented, firstly with the tibia and lastly with the femoral component (Figure 6). Standard closure and dressing are then performed. Pre- and postoperative X-rays of a case example are shown in Figure 7. The surgical technique video is provided at the end of the paper as Supplementary Materials.

### 2.8. Postoperative Protocol

In our institution, postoperative management after custom TKA follows the same standardized protocol as for conventional primary TKA. Patients are allowed full weight-bearing as tolerated from the first postoperative day, with early active and assisted range-of-motion exercises and supervised quadriceps activation. Walking aids are used as needed in the early phase, and outpatient physiotherapy is continued for approximately 6–12 weeks, focusing on recovery of flexion–extension, gait training, and muscle strengthening. No specific modifications of the rehabilitation pathway are required because of the use of individualized implants.

## 3. Discussion

The Origin^®^ system offers several advantages related to the increasingly popular concept of “individualized TKA”. Through precise restoration of individual anatomy, individualized implants aim at overcoming the “one size fits all” dogma characterizing most traditional alignment strategies, which have been frequently associated with iatrogenic laxity or stiffness [8]. The customization of bone cuts and implants allows us to minimize implant thickness and weight, as well as reducing the extent of bone resection required [9]. Notably, technologies such as custom cutting guides or navigation systems, while generally used with standard implants, have not consistently demonstrated significant benefits in terms of patient outcomes or satisfaction [10,11]. Therefore, it has been proposed that TKA outcomes may be significantly improved by focusing on three key elements: the development of a personalized alignment strategy, the adoption of advanced technologies such as robotics for greater surgical precision, and the use of custom implants to restore native knee anatomy [12].

Individualized TKA offers several advantages to the surgeon. First, it simplifies the surgical process by conserving or restoring native anatomy, which helps mitigate many surgical challenges [13]. For example, femoral and tibial rotations are pre-adjusted during the design phase, reducing the need for intraoperative adjustments, which are often manual and not totally precise. This also simplifies ligament balancing, particularly at mid-flexion, by preserving the natural condylar curvature radii and joint line obliquity. Additionally, individualized implants eliminate the need for size adjustments, as they are precisely tailored to fit the patient’s bone structure. Second, preoperative planning of alignment and implant positioning provides greater intraoperative guidance and security for the surgeon, ensuring a higher degree of precision [4]. This technology is especially advantageous in complex cases, such as patients with post-traumatic extra-articular deformities, where anatomical corrections are more easily achieved [14]; patients with unextractable hardware from previous surgeries, where the design can prevent impingement; patients with multi-operated or previously infected bones, where minimal bone manipulation reduces the risk of complications; and patients with extreme anatomical variations, where standard TKAs may be inadequate or challenging to implant. Nonetheless, several studies have shown that customized TKA has demonstrated better clinical results and patients’ satisfaction compared to conventional implants [15,16]. However, the use of individualized TKA implants has some limitations. These include increased costs and time for production, higher radiation exposure due to the preoperative CT scan needed for preoperative planning, and the limited evidence base compared to more popular implants. Additionally, they are mainly conceived for moderate deformity in the setting of osteoarthritis, with more significant pathologies, such as significant patellofemoral maltracking, still requiring conventional, off-the-shelf implants with additional soft tissue procedures.

## 4. Conclusions

The Origin^®^ individualized TKA system represents a significant advancement in knee arthroplasty by providing a tailored approach to patient care. Future studies are needed to further evaluate the long-term outcomes and cost-effectiveness of this personalized system compared to conventional TKA approaches.

## Data Availability

No new data were created or analyzed in this study.

## Supplementary Materials

The following supporting information can be downloaded at https://www.mdpi.com/article/10.3390/jcm15093356/s1: Video S1: Surgical Technique Video.

## Abbreviations

TKA	Total knee arthroplasty
CR	Cruciate-retaining
PS	Posterior-stabilized
ROM	Range of motion
ACL	Anterior cruciate ligament
PCL	Posterior cruciate ligament

## References

[1] Nham F.H., Patel I., Zalikha A.K., El-Othmani M.M. (2023). Epidemiology of primary and revision total knee arthroplasty: Analysis of demographics, comorbidities and outcomes from the national inpatient sample. Arthroplasty.

[2] Karasavvidis T., Pagan Moldenhauer C.A., Haddad F.S., Hirschmann M.T., Pagnano M.W., Vigdorchik J.M. (2023). Current Concepts in Alignment in Total Knee Arthroplasty. J. Arthroplast..

[3] Hirschmann M.T., von Eisenhart-Rothe R., Graichen H., Vendittoli P.A., Chen A.F., Lustig S., Leal J., Tibesku C., Bonnin M. (2024). Neutrality, normality, abnormality and pathology in coronal knee alignment: Why and how should we define it in the era of personalised medicine?. Knee Surg. Sports Traumatol. Arthrosc..

[4] Saffarini M., Hirschmann M.T., Bonnin M. (2023). Personalisation and customisation in total knee arthroplasty: The paradox of custom knee implants. Knee Surg. Sports Traumatol. Arthrosc..

[5] Vendittoli P.-A., Martinov S., Blakeney W.G. (2021). Restricted Kinematic Alignment, the Fundamentals, and Clinical Applications. Front. Surg..

[6] Victor J., Vermue H. (2021). Custom TKA: What to expect and where do we stand today?. Arch. Orthop. Trauma Surg..

[7] Iwano T., Kurosawa H., Tokuyama H., Hoshikawa Y. (1990). Roentgenographic and Clinical Findings of Patellofemoral Osteoarthrosis. Clin. Orthop. Relat. Res..

[8] Beckmann J., Meier M.K., Benignus C., Hecker A., Thienpont E. (2021). Contemporary knee arthroplasty: One fits all or time for diversity?. Arch. Orthop. Trauma Surg..

[9] Patil S., Bunn A., Bugbee W.D., Colwell C.W., D’Lima D.D. (2015). Patient-specific implants with custom cutting blocks better approximate natural knee kinematics than standard TKA without custom cutting blocks. Knee.

[10] Chughtai M., Khlopas A., Davidson I.U., Yakubek G.A., Stearns K.L., Mont M.A. (2017). Custom cutting guides in total knee arthroplasty. Ann. Transl. Med..

[11] Shah S.M. (2021). After 25 years of computer-navigated total knee arthroplasty, where do we stand today?. Arthroplasty.

[12] Sappey-Marinier E., Tibesku C., Selmi T.A.S., Bonnin M. (2020). Custom Total Knee Arthroplasty. Personalized Hip and Knee Joint Replacement.

[13] Bonnin M.P., Beckers L., Leon A., Chauveau J., Müller J.H., Tibesku C.O., Aït-Si-Selmi T. (2020). Custom total knee arthroplasty facilitates restoration of constitutional coronal alignment. Knee Surg. Sports Traumatol. Arthrosc..

[14] Daxhelet J., Aït-Si-Selmi T., Müller J.H., Saffarini M., Ratano S., Bondoux L., Mihov K., Bonnin M.P. (2021). Custom TKA enables adequate realignment with minimal ligament release and grants satisfactory outcomes in knees that had prior osteotomies or extra-articular fracture sequelae. Knee Surg. Sports Traumatol. Arthrosc..

[15] Zeh A., Gehler V., Gutteck N., Beckmann J., Brill R., Wohlrab D. (2022). Superior clinical results and higher satisfaction after customized compared with conventional TKA. Acta Orthop. Belg..

[16] Gousopoulos L., Dobbelaere A., Ratano S., Bondoux L., Müller J.H., Dubreuil S., Saffarini M., Tibesku C.O., Aït-Si-Selmi T., Bonnin M.P. (2023). Custom total knee arthroplasty combined with personalised alignment grants 94% patient satisfaction at minimum follow-up of 2 years. Knee Surg. Sports Traumatol. Arthrosc..

